# A Purge-and-Trap Gas Chromatography–Mass Spectrometry Method for the Quantitative Determination of Six Haloacetonitriles in Drinking Water

**DOI:** 10.3390/toxics14030214

**Published:** 2026-02-28

**Authors:** Yuan Wang, Yuyan Liu, Jiafu Li, Xueqin Huang, Junling Li, Xiaojun Liang

**Affiliations:** 1 Center for Disease Control and Prevention of Kunshan, Kunshan 215300, China; yuanyuan85922@163.com; 2 School of Public Health, Suzhou Medical College, Soochow University, Suzhou 215127, China; jiafuli@suda.edu.cn (J.L.); 20235247071@stu.suda.edu.cn (X.H.); tljunling@163.com (J.L.); 3 School of Nursing and Healthcare, Shandong Vocational University of Foreign Affairs, Rushan 264504, China; 4 National Institute for Radiological Protection, Chinese Center for Disease Control and Prevention, Beijing 100050, China

**Keywords:** haloacetonitriles, drinking water, GC-MS, purge-and-trap

## Abstract

Haloacetonitriles (HANs), toxic disinfection by-products, are unregulated in China, with no standardized analytical methods. This study established a simultaneous quantitative method for six typical HANs in drinking water using an optimized purge-and-trap gas chromatography–mass spectrometry (P&T-GC/MS) system. Key parameters, including sorbent trap selection, purge time, and moisture control settings, were systematically optimized. The OI No. 7 trap and a 13 min purge time were selected to maximize sensitivity while minimizing moisture interference. Under optimal conditions, all target analytes showed good linearity (R^2^ > 0.999). The method detection limits (LODs) ranged from 0.007 to 0.202 μg/L, and the limits of quantitation (LOQs) ranged from 0.2 to 2.0 μg/L. Average spiked recoveries in tap water were 89.5–111.0%, with relative standard deviations (RSDs) below 5% (*n* = 7). A core optimization was omitting pH adjustment and ascorbic acid quenching to avoid non-target degradation of brominated HANs and ensure accurate in situ concentration determination. Application to 16 Kunshan tap water samples showed total HAN concentrations of 0.59–3.03 μg/L (average: 1.62 μg/L), dominated by bromochloroacetonitrile (BCAN) and dibromoacetonitrile (DBAN). Process analysis indicated significant synergistic HAN removal by sand filtration and activated carbon, while chloramination significantly increased brominated HANs via enhanced bromination. This efficient, sensitive P&T-GC/MS method is suitable for trace HAN monitoring and provides technical support for HAN control in water treatment.

## 1. Introduction

Drinking water safety is a crucial cornerstone of the public health system [1]. Disinfection by-products (DBPs), as harmful substances generated during water treatment processes, have become a key focus of drinking water quality monitoring worldwide [2,3,4,5,6]. Among the more than 700 identified DBPs, haloacetonitriles (HANs) exhibit significantly higher cytotoxicity and genotoxicity than conventional carbon-containing DBPs due to their unique nitrogen-containing structure [4,7,8,9,10,11] and have attracted widespread attention from the academic community and regulatory authorities in recent years. Toxicological studies have shown that typical HANs, such as dichloroacetonitrile (DCAN) and bromochloroacetonitrile (BCAN), may induce carcinogenic risks even at the µg/L concentration level, and their toxicity contribution rate significantly exceeds that of regulated trihalomethanes [3,11,12,13]. However, due to the low concentration and chemical instability of HANs in drinking water, existing analytical methods still have obvious deficiencies in terms of sensitivity, selectivity, and operational convenience [14,15].

Current conventional detection technologies face multiple challenges: liquid–liquid extraction requires the consumption of a large amount of organic solvents and involves cumbersome pretreatment steps [16]; headspace injection has insufficient detection sensitivity for low-boiling-point HANs [17]; and, although solid-phase microextraction can reduce solvent usage, it has problems such as short fiber lifetime and poor batch reproducibility [18,19]. Despite the development of various chromatographic detection methods by researchers in recent years, these methods either rely on special chromatographic columns (e.g., Rtx-200) or make achieving simultaneous and accurate quantification of multiple HANs difficult, thus limiting their application in practical water quality monitoring [20,21].

To address the aforementioned technical bottlenecks, this study developed a quantitative analytical method based on purge-and-trap gas chromatography–mass spectrometry (P&T-GC/MS), enabling efficient detection of six key HANs (chloroacetonitrile, dichloroacetonitrile, bromoacetonitrile, bromochloroacetonitrile, dibromoacetonitrile, and iodoacetonitrile) in drinking water. This method uses a universal DB-624 chromatographic column to optimize separation efficiency and employs purge-and-trap technology for fully automated sample pretreatment. This approach not only significantly reduces organic solvent consumption but also achieves method detection limits (0.007–0.202 μg/L) and precision levels (RSD < 5.0%) that are superior to those of current standard methods. The innovation of this study lies in establishing an analytical platform that balances high sensitivity and practicality, providing new technical support for drinking water safety monitoring and a scientific basis for the formulation of DBP control strategies in water treatment processes.

## 2. Methods

### 2.1. Instruments and Chemical Reagents

TRACE 1300 gas chromatograph (Thermo Fisher Scientific, Waltham, MA, USA), ISQ 7000 mass spectrometer (Thermo Fisher Scientific, Waltham, MA, USA), equipped with a DB-624 capillary column (60 m × 0.25 mm × 1.4 μm; Agilent Technologies, Santa Clara, CA, USA). Eclipse 4760 purge-and-trap concentrator (OI Analytical, College Station, TX, USA), haloacetonitrile (HAN) analytical standards (chloroacetonitrile, C_2_H_2_ClN; dichloroacetonitrile, C_2_HCl_2_N; bromoacetonitrile, C_2_H_2_BrN; dibromoacetonitrile, C_2_HBr_2_N; bromochloroacetonitrile, C_2_HBrClN; iodoacetonitrile, C_2_H_2_IN), certified reference materials (First Standard, Tianjin, China), 1 mg/mL concentration, analytical grade reagents.

### 2.2. Preparation of Standard Solutions and Calibration

Individual stock solutions (1 mg/mL) were prepared by dissolving 0.01 g of each HAN standard in 10 mL of acetonitrile and stored at −20 °C (stable for 24 months). A mixed intermediate standard solution and a mixed working solution were prepared by diluting the stocks in acetonitrile to specific concentrations in the µg/mL range, as summarized in Table 1.

For the calibration, a five-point calibration curve was constructed using the external standard method. Calibration standards were prepared by spiking 10, 20, 40, 80, and 100 μL of the mixed working solution into 100 mL of ultrapure water to achieve the final concentration levels (µg/L). These standards were analyzed following the same P&T-GC/MS procedure as the samples. The calibration curves were established by plotting the peak areas of the quantitation ions against their corresponding mass concentrations.

### 2.3. Sample Collection and Preparation

A total of 16 tap water samples (500 mL each) were collected from the main urban areas of Kunshan City. The source water for the studied treatment plants mainly originates from Kulei Lake and the Yangtze River. Kulei Lake is a lacustrine-type water source located in Bacheng Town, whereas the Yangtze River is a river-type source located near Changshu. Both are classified as Class II surface water sources. The Yangtze River water is transported to Kunshan through a DN2200 pipeline, where it is mixed proportionally with Kulei Lake water before entering the treatment plant. Chloramination is used as the primary disinfection process.

Prior to sampling, tap water was flushed for 3 min, and amber glass bottles were rinsed three times with tap water. The influence of sampling bottles and transportation on analytical results was assessed using ultrapure water. Collected tap water samples were transported on ice to the laboratory and analyzed within 12 h.

In addition, seven water samples (1000 mL each) were collected from the outlet of each treatment unit at the No. 3 Water Treatment Plant in Kunshan City. Each sample was divided into two groups: one was quenched with ascorbic acid, and the other was left untreated. Forty milliliters of each water sample was transferred into headspace vials and analyzed for HANs. Milli-Q water was used as the field and trip blank.

### 2.4. Purge-and-Trap Gas Chromatography–Mass Spectrometry (P&T-GC/MS) Analysis

Sample analyses were performed using the TRACE 1300 GC coupled with an ISQ 7000 MS (both from Thermo Fisher Scientific, Waltham, MA, USA), equipped with an EI source, and an Eclipse 4760 P&T concentrator (OI Analytical, College Station, TX, USA) featuring an autosampler, a 25 mL purge vessel, and a 25 mL sample loop. Chromatographic separation was conducted in the multiramp programmable oven of the TRACE 1300 system using a DB-624 capillary column (60 m × 0.25 mm × 1.4 μm) or an equivalent column.

The oven temperature program was optimized as follows: the initial temperature of 40 °C was held for 2 min, increased at 5 °C/min to 120 °C, and then raised at 10 °C/min to 200 °C (held for 0 min). Ultra-high purity helium (≥99.999%) served as the carrier gas at a constant flow rate of 1.0 mL/min. The injector temperature was maintained at 220 °C, and injections were made in split mode (split ratio 5:1). The mass spectrometer operated in electron ionization (EI) mode at 70 eV, with the ion source and transfer line temperatures set at 280 °C and 250 °C, respectively.

Purge-and-trap conditions were set as follows: a No. 7 trap (Tenax TA only, Part No. 227348, O.I. Analytical, College Station, TX, USA) was employed; the purge step was carried out at an ambient temperature for 13 min, with a purge volume of 25 mL at a purge flow rate of 40 mL/min. Desorption was performed at 180 °C for 1 min, followed by a bake step at 200 °C for 15 min. The integrated Cyclone water management system was operated at 120 °C, 120 °C, and 240 °C during the purge, desorption, and bake stages, respectively, to selectively remove moisture while preserving target analytes. Samples were analyzed in selected ion monitoring (SIM) mode. Detailed ion information and method performance for the six HANs are summarized in Table 2 [22].

### 2.5. Data Processing and Statistical Analysis

Data processing and concentration calculations for the six HANs were performed using Microsoft Office Excel. Descriptive statistics, including the mean and standard deviation (SD), were employed to summarize the concentration levels across the water samples. All graphical representations and data visualizations were generated using GraphPad Prism (version 10.6, GraphPad Software, San Diego, CA, USA).

### 2.6. Quality Assurance and Quality Control (QA/QC)

To ensure analytical reliability, QA/QC procedures were strictly implemented. The limit of detection (LOD) and limit of quantification (LOQ) were determined by seven replicate analyses (*n* = 7) of ultrapure water samples spiked at the lowest calibration level. According to the standard statistical approach, the LOD was calculated as 3.14 × S and the LOQ as 10 × S, where S represents the standard deviation of the seven replicates. Method accuracy and precision were evaluated through recovery tests by spiking tap water samples at three concentration levels (low, medium, and high), with six replicates for each level. The recovery was calculated as the percentage of the detected concentration relative to the spiked concentration, while the relative standard deviation (RSD) was used to assess precision. Furthermore, laboratory and field blanks were analyzed for each batch of samples to monitor potential cross-contamination; no target HANs were detected in any blank samples. Additionally, a check standard and a blank were analyzed after every 20 samples to ensure the stability of the instrumental response.

## 3. Results and Discussion

### 3.1. Method Performance and Validation

**Figure 1:**
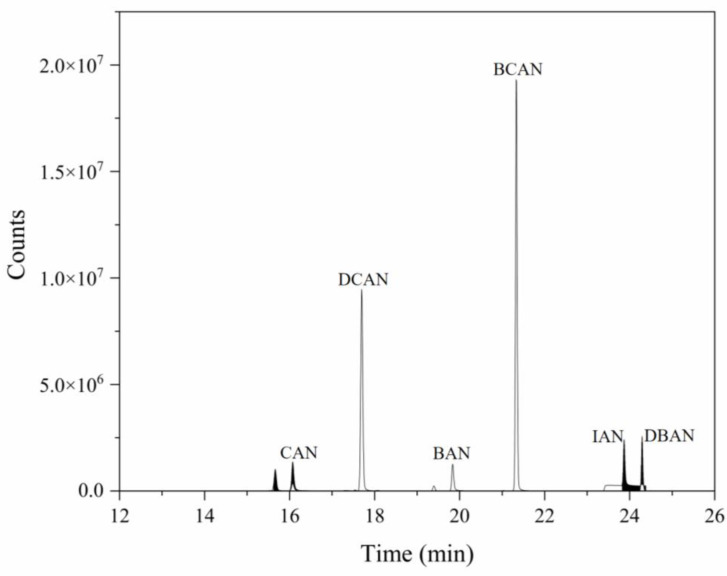
TIC map of 6 HANs (spiked levels = 5 μg/L).

As shown in Figure 1, all six HANs exhibited highly symmetrical chromatographic peaks with widths less than 10 s, indicating that the optimized column and temperature program enabled complete separation. The performance characteristics of the P&T-GC/MS method, derived from the experimental data, are summarized in Table 2.

The method demonstrated high sensitivity, with LODs ranging from 0.007 to 0.202 μg/L and LOQs from 0.2 to 2.0 μg/L for all analytes. These values are well below the regulatory limits and typical environmental concentrations, confirming the suitability of the method for trace analysis. The average spike recoveries for the six HANs at three different levels (low, medium, and high) ranged from 89.5% to 111.0% (Table 2). The method precision, expressed as RSD, was below 5.0% for all analytes (*n* = 7), demonstrating excellent accuracy and reproducibility.

Furthermore, the external standard calibration curves showed excellent linearity. As detailed in Table 3, the correlation coefficients (R^2^) for all six HANs exceeded 0.9990 within their respective linear ranges, ensuring reliable quantification across the studied concentration gradients.

### 3.2. Optimization of Analytical Conditions

#### 3.2.1. Selection of the Sorbent Trap

The adsorption performance of the sorbent trap is a critical factor influencing the enrichment efficiency and detection sensitivity of target analytes. In this study, the trapping efficiencies of the OI No. 10 trap (Tenax/silica gel/carbon molecular sieve) and the OI No. 7 trap (Tenax TA only, Part No. 227348) were evaluated for six HANs at a concentration of 5.0 μg/L.

**Figure 2:**
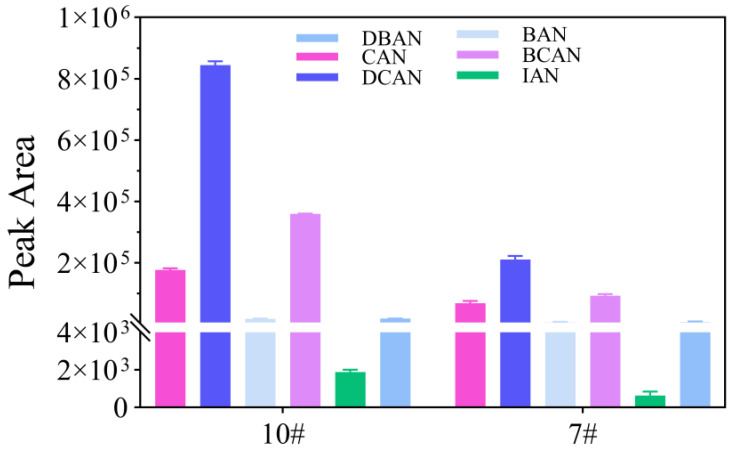
Collection efficiency of different sorbent traps for HANs (*n* = 3). Experimental conditions: purge time: 15 min; cyclone water management system: purge at 120 °C, desorption at 120 °C, and bake at 240 °C. The Y-axis represents the peak area (counts), and data are presented as mean ± SD (*n* = 3). (#7: OI No. 7 trap; #10: OI No. 10 trap).

As illustrated in Figure 2, although the No. 10 trap exhibited higher peak areas for CAN, DCAN, and BCAN, the No. 7 trap provided a more balanced and stable response across all six HANs, particularly for challenging analytes such as IAN and BAN. The observed differences are primarily attributed to the filler compositions and their corresponding adsorption mechanisms. Tenax TA, the single packing material in the No. 7 trap, is a nonpolar to slightly polar porous polymer known for its excellent thermal stability and high hydrophobicity [23]. These properties facilitate rapid and complete desorption while minimizing water interference, ensuring long-term system stability.

In contrast, the inclusion of silica gel and carbon molecular sieves in the No. 10 trap, while providing a larger surface area for increased capacity, may lead to excessively strong retentive forces [24,25]. This can result in incomplete desorption or carryover effects for certain nitrogen-containing disinfection by-products (N-DBPs) under standard P&T conditions. Because the No. 7 sorbent trap offered superior robustness for challenging HANs, it was selected as the optimal trap for this study.

#### 3.2.2. Optimization of Purge Time

**Figure 3:**
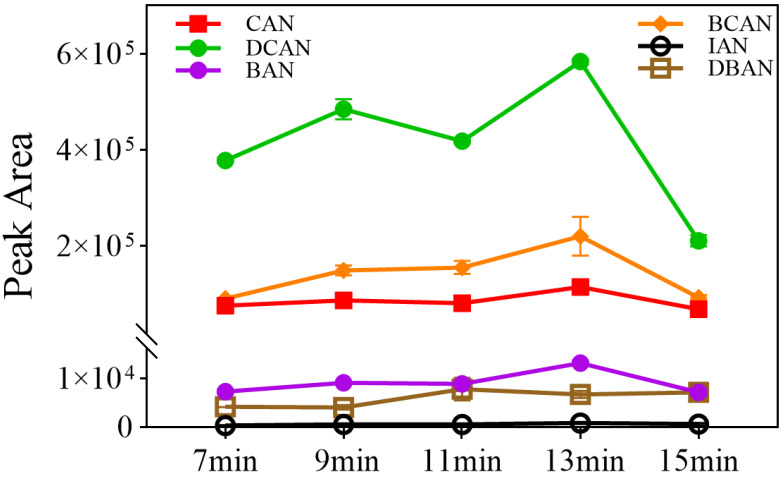
Effect of collection time of purge-and-trap on response values of HANs (*n* = 3).

This experiment investigated the response intensities of 5.0 µg/L HANs under purge times of 7, 9, 11, 13, and 15 min (*n* = 3). As shown in Figure 3, the response intensity of each HAN first increased and then decreased with the extension of purge time. The maximum response values for most HANs were achieved at 13 min, while the response for IAN remained relatively stable at a lower level.

The observed variation is related to the balance between stripping efficiency, moisture interference, and analyte breakthrough. If the purge time is too short (e.g., 7 min), the target compounds cannot be fully stripped from the water sample, resulting in low response signals. Conversely, exceeding the optimal time (e.g., 15 min) led to a significant decrease in sensitivity. This decline is likely due to the excessive accumulation of moisture that enters the trap and interferes with the adsorption and subsequent MS detection. Furthermore, it may involve analyte breakthrough, where the continuous flow of purge gas exceeds the retentive capacity of the sorbent trap, causing target analytes to be carried out of the trap before the desorption stage begins [26,27]. Therefore, a purge time of 13 min was selected as the optimal condition to maximize stripping efficiency while effectively avoiding breakthrough- and moisture-related suppression.

#### 3.2.3. Optimization of Purge Conditions

The performance of the Moisture Control System (MCS) significantly influences both detection sensitivity and system stability. In this study, the response behaviors of HANs were compared under two settings: “With MCS” (120 °C purge temperature + 3 min dry purge) and “Without MCS” (ambient temperature without dry purge).

**Figure 4:**
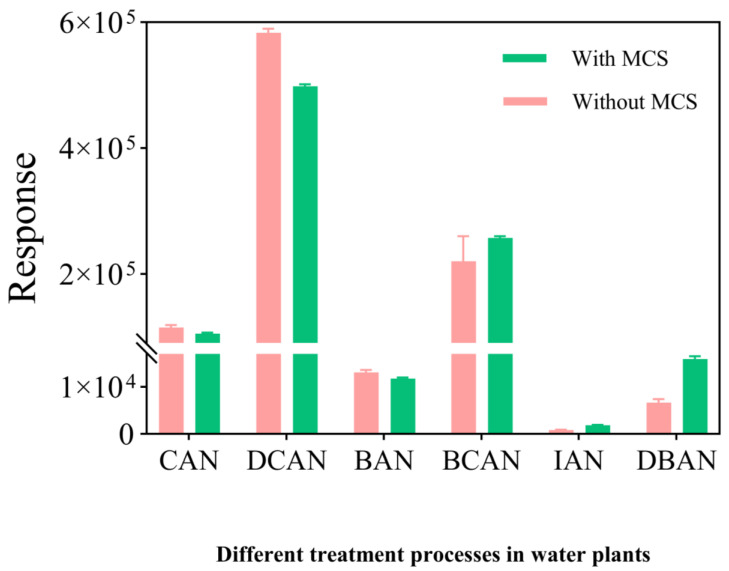
Influence of heating conditions of purge-and-trap on sensitivity of HANs (*n* = 3).

As shown in Figure 4, the target compounds exhibited distinct responses to the moisture control settings. CAN and DCAN showed higher peak areas without a MCS, whereas the implementation of the MCS led to a decrease in their response. Conversely, the sensitivities of IAN and DBAN were improved by approximately 2-fold and 1-fold, respectively, under the MCS-enabled conditions.

The observed variations are primarily attributed to the balance between moisture removal and analyte retention. During the purge stage, the MCS is heated (120 °C) to prevent early condensation of analytes [28]. Following a 3 min reverse dry purge, the system enters the desorption stage, where moisture is condensed and discharged. While this effectively reduces water interference for the chromatographic system [29], highly polar compounds like CAN and DCAN are prone to co-precipitation with the condensed water, leading to their reduced sensitivity. However, for less polar and less volatile compounds such as IAN and DBAN, the removal of water vapor significantly enhances signal stability and overall detection performance [30]. Consequently, the MCS-enabled protocol was selected to ensure the long-term robustness of the method for multi-component HAN analysis.

#### 3.2.4. Effect of Residual Chlorine Quenching Agent

**Figure 5:**
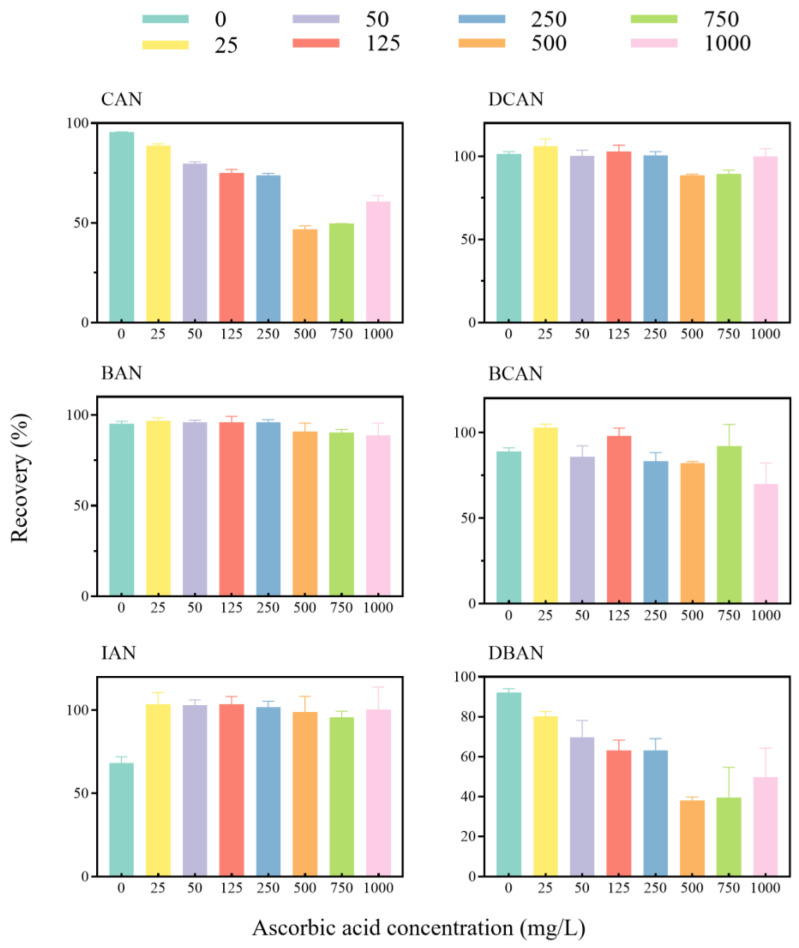
Effect of residual chlorine quencher on HAN detection (*n* = 3).

To evaluate the effect of residual chlorine quenching agents on the six HANs, tap water samples containing 1.0 mg/L free residual chlorine were treated with ascorbic acid at concentrations from 0 to 1000 mg/L (*n* = 3). As shown in Figure 5, the addition of ascorbic acid resulted in distinct stability profiles among the target compounds. DCAN and BAN remained highly stable across all concentrations, while IAN exhibited low recovery (68.2%) without ascorbic acid but stabilized at approximately 100% upon the addition of quencher. CAN and DBAN displayed V-shaped trends, with recoveries decreasing to minimum values (46.8% and 38.0%, respectively) at 500 mg/L and then recovering at higher concentrations, whereas BCAN fluctuated moderately within 75–100%.

These results indicate that, although ascorbic acid can eliminate oxidative interference from residual chlorine [31], its strong reducibility may induce non-targeted reactions and degradation of certain HANs. Given that 1.0 mg/L residual chlorine did not cause significant oxidative loss for most analytes, the addition of ascorbic acid introduced greater instability than protection [31]. Therefore, to achieve consistent and reliable recoveries for all six HANs, no residual chlorine quencher was used in the subsequent analytical procedures.

#### 3.2.5. Effect of Sample pH on the Determination of Six Haloacetonitriles

**Figure 6:**
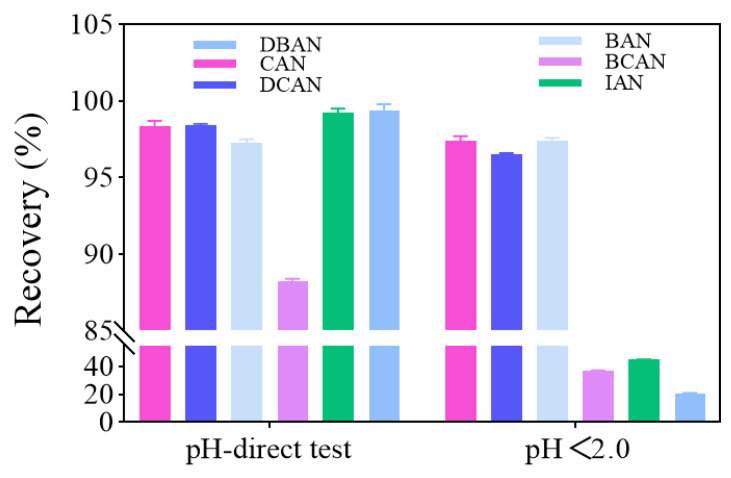
Influence of water sample pH on HAN detection (*n* = 3).

The influence of sample pH on the recovery of six HANs was evaluated by comparing spiked tap water (μg/L) at its natural pH with a sample adjusted to pH < 2 with hydrochloric acid. The results are illustrated in Figure 6. Without pH adjustment, all six HANs exhibited excellent recoveries: CAN, DCAN, and BAN reached 98.7%, 98.5%, and 97.5%, respectively, while BCAN, IAN, and DBAN remained within the ideal range of 88.4–99.8%. However, upon acidification to pH < 2, while the recoveries of CAN, DCAN, and BAN remained stable (96.6–97.7%), those of BCAN, IAN, and DBAN plummeted to 20.6–44.5%, failing to meet quantitative requirements.

This significant discrepancy is attributed to the synergistic effect of the chemical structure of specific HANs and the water matrix [32,33,34]. CAN, DCAN, and BAN, which contain single chlorine or bromine substituents, possess higher C-halogen bond energies and resist hydrolysis even under strongly acidic conditions. In contrast, the mixed bromine–chlorine substitution in BCAN, iodine substitution in IAN, and dibromine substitution in DBAN result in uneven molecular polarity or reduced bond energies. These structural features trigger rapid hydrolysis or dehalogenation under strong acidity, leading to substantial analyte loss [15]. Consequently, to ensure the stability and accuracy of all six HANs, direct determination without pH adjustment was adopted as the optimized protocol.

### 3.3. Determination of Haloacetonitriles in Drinking Water Samples

**Figure 7:**
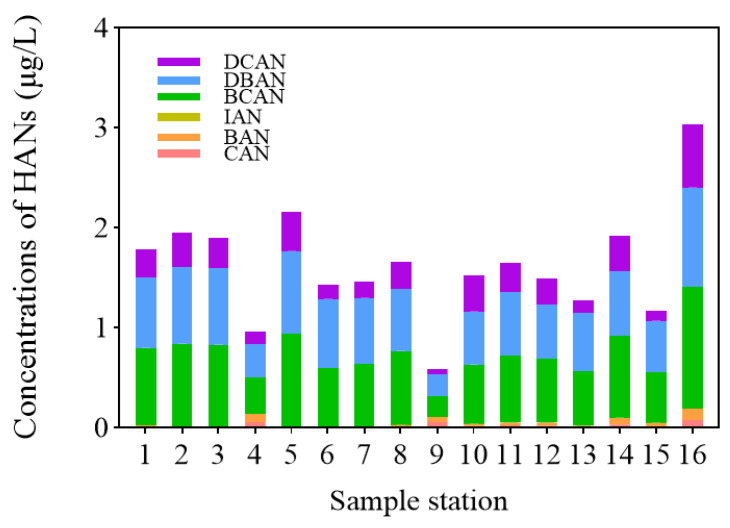
Concentration of HANs in home drinking water of Kunshan. (Note: IAN was not detected in the samples).

The presence of six HANs in 16 tap water samples from Kunshan was analyzed under the optimized P&T-GC/MS conditions. Target concentrations were quantified using the external standard method based on the established calibration curves. As illustrated in Figure 7, various HANs were detected in all samples, with total concentrations ranging from 0.59 to 3.03 μg/L (average: 1.62 μg/L).

Among the detected species, BCAN exhibited the highest average concentration (0.68 μg/L), followed by DBAN (0.63 μg/L). The average concentrations for other HANs were 0.26 μg/L for DCAN, 0.03 μg/L for BAN, and 0.02 μg/L for CAN, while IAN was not detected in any samples. Given that HANs are highly toxic nitrogenous DBPs (N-DBPs), with cytotoxicity levels 2–3 orders of magnitude higher than those of trihalomethanes [7], monitoring their prevalence is of critical importance for ensuring drinking water safety.

### 3.4. Method Performance Evaluation

As summarized in Table 4, the analytical method developed in this study exhibited competitive performance across multiple key performance indicators. The method precision was favorable, with the relative standard deviations (RSDs) for all target analytes maintained below 5.0%, which is comparable to or better than many previously reported methods. The method sensitivity was particularly remarkable: the LODs ranged from 0.007 to 0.202 μg/L, and the LOQs ranged from 0.2 to 2.0 μg/L, enabling the determination of HANs at low concentrations. In terms of accuracy, the spike recoveries remained consistently within 89.5–111.0%, indicating acceptable resistance to matrix interference.

Compared with existing analytical techniques, this method enables the simultaneous quantification of six HAN species with a minimum detectable concentration of 0.007 μg/L. By optimizing purge-and-trap pretreatment and employing GC-MS detection, the proposed method provides a reliable analytical tool for trace-level monitoring of DBPs in drinking water.

### 3.5. Influence of Different Water Treatment Processes on the Formation of Haloacetonitriles (HANs)

**Figure 8:**
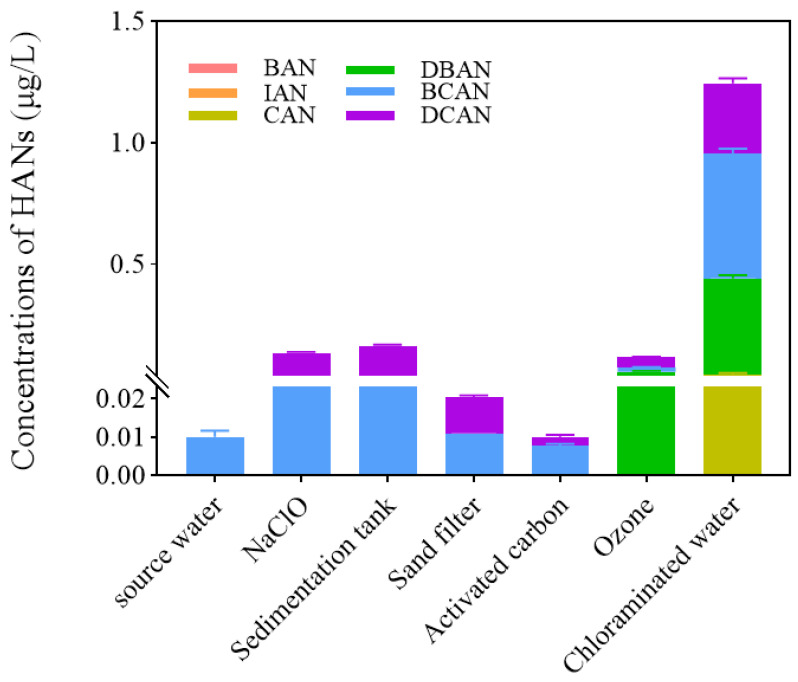
Impact of drinking water treatment process on HAN concentration. (Note: IAN and BAN = N.D. in all samples).

The concentration and speciation of haloacetonitriles (HANs) fluctuated significantly across different treatment stages (Figure 8). While source water contained only trace HANs [5], sodium hypochlorite (NaClO) disinfection was associated with a sharp increase, primarily in DCAN. This phenomenon is hypothesized to arise from the lower activation energy and faster diffusion of chlorine species reacting with nitrogenous precursors [45,46]. Concurrently, the activation of potential trace bromide (Br^−^) into reactive bromine (e.g., BrCl) may have promoted the formation of BCAN [34,49,50]. Flocculation and sedimentation led to a slight rebound in HANs, which could be attributed to precursors aggregating in localized high-density areas, thus increasing contact with residual disinfectants [14].

In contrast, sand filtration and activated carbon achieved substantial HAN removal [47]. This synergy potentially relies on physical interception by sand and the porous structure of activated carbon (surface area 1000–1500 m^2^/g), which may adsorb nitrile groups via van der Waals forces and hydrogen bonding [9,14,51,52,53]. However, concentrations rebounded following ozonation, specifically in dibromoacetonitrile (DBAN) [48]. It is speculated that ozone degrades complex organic bromine into reactive small-molecule precursors and accelerates Br^−^ activation, while the degradation rate of DBAN by ozone remains relatively slow [54,55,56]. The final chloramination stage saw HANs reach peak levels, with prominent increases in brominated species like DBAN and BCAN. The low oxidation potential of chloramine is reported to favor the formation of electrophilic BrCl, which, combined with extended contact times, potentially promotes the massive generation of brominated HANs [57,58].

**Figure 9:**
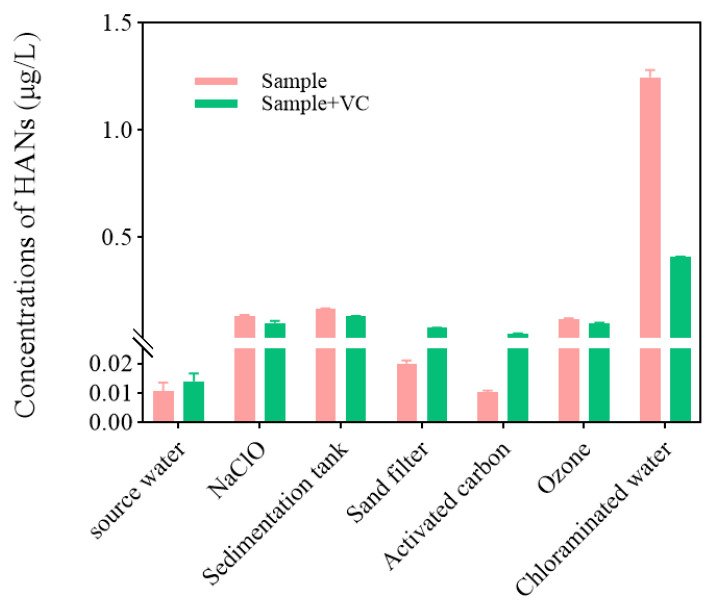
Impact of Vc on HANs concentration in water samples.

This study also evaluated the impact of ascorbic acid (Vc) on detection accuracy across the process (Figure 9). In most stages, Vc addition yielded results similar to direct measurement, except for the activated carbon effluent—where Vc potentially inactivated adsorption sites—and the final chloramination effluent. In the latter, Vc addition caused the detected HANs to plummet from >1.2 μg/L to <0.4 μg/L. This discrepancy arises because Vc rapidly reduces residual active halogens [59], halting further HAN formation post-sampling; more critically, its strong reducibility may degrade the nitrile structure of existing brominated/iodinated HANs. Consequently, to accurately characterize real-time HAN concentrations across all process stages, no ascorbic acid should be added during the analytical procedure.

## 4. Conclusions

This study successfully developed and validated a high-sensitivity P&T-GC/MS method for the simultaneous determination of six haloacetonitriles (HANs) in drinking water. The method exhibited excellent sensitivity and precision, with method detection limits (LODs) of 0.007–0.202 μg/L and limits of quantitation (LOQs) of 0.2 to 2.0 μg/L, both well below typical environmental concentrations. The optimized protocol achieved satisfactory recoveries of 89.5–111.0% and high reproducibility with relative standard deviations (RSDs) below 5%, without requiring complex manual pretreatment. Regarding condition optimization, the OI No. 7 sorbent trap combined with a 13 min purge time provided the optimal balance between extraction efficiency and system stability, and the Moisture Control System (MCS), operated at 120 °C, significantly improved the signal response for less volatile compounds such as IAN and DBAN. With respect to chemical stability and pretreatment, this study confirmed that the addition of ascorbic acid as a chlorine quencher or adjusting the pH to below 2.0 caused obvious degradation of brominated and iodinated HANs, including BCAN, IAN, and DBAN; therefore, direct analysis without pH adjustment or chemical quenching is recommended for accurate in situ concentration determination. In terms of practical application to drinking water in Kunshan, the average total HAN concentration was 1.62 μg/L, dominated by BCAN and DBAN; sand filtration and activated carbon treatment effectively removed HANs, whereas chloramination was identified as a major contributor to the formation of brominated HANs. Overall, this automated P&T-GC/MS method provides a reliable and powerful tool for routine water quality monitoring and offers a solid scientific basis for the control of nitrogenous disinfection by-products (N-DBPs) and the assurance of drinking water safety.

## Figures and Tables

**Table 1:** Concentrations of mixed standard solutions and calibration levels.

HANs	Intermediate (µg/mL)	Working (µg/mL)	Calibration Range (µg/L)
CAN	20.0	2.0	0.2, 0.4, 0.8, 1.6, 2.0
DCAN	20.0	2.0	0.2, 0.4, 0.8, 1.6, 2.0
BAN	100	10.0	1.0, 2.0, 4.0, 8.0, 10.0
BCAN	100	10.0	1.0, 2.0, 4.0, 8.0, 10.0
IAN	200	20.0	2.0, 4.0, 8.0, 16.0, 20.0
DBAN	100	10.0	1.0, 2.0, 4.0, 8.0, 10.0

**Table 2:** Parameters for HAN analysis (*n* = 7).

HANs	Retention Time (min)	Quant/Qual Ion (m/z)	LOD (μg/L)	LOQ (μg/L)	Recovery Range (%)	RSD (%)
CAN	16.1	75.0/77.0	0.007	0.2	96.7–110.0	1.3
DCAN	17.7	82.0/75.0	0.011	0.2	89.5–102.0	1.0
BAN	19.9	79.0/119.0	0.056	1.0	96.5–111.0	1.9
BCAN	21.4	74.0/76.0	0.048	1.0	91.1–98.0	1.8
IAN	23.9	167.0/127.0,40.0	0.202	2.0	98.6–103.0	3.2
DBAN	24.3	120.0/199.0	0.042	1.0	93.1–104.0	4.8

Note: The LOQs were defined as the lowest concentration levels of the validated calibration curves to ensure quantitative reliability.

**Table 3:** Linear range and correlation coefficient of haloacetonitrile in drinking water (*n* = 5).

HANs	Regression Equation	Linear Range (μg/L)	R^2^
CAN	y = 1.6 × 104x + 1.2 × 10^3^	0.2–2.0	0.9995
DCAN	y = 4.1 × 104x − 2.2 × 10^3^	0.2–2.0	0.9995
BAN	y = 2.5 × 103x − 7.4 × 10^2^	1.0–10.0	0.9999
BCAN	y = 2.8 × 104x − 1.7 × 10^4^	1.0–10.0	0.9996
IAN	y = 6.5 × 102x − 7.3 × 10^2^	2.0–20.0	0.9994
DBAN	y = 4.0 × 103x − 3.4 × 10^3^	1.0–10.0	0.9994

**Table 4:** A Comparison between the present method and the published method.

Pre-Treatment	Instrument	LOD (10^−3^ µg/L)	Rec. (%)	RSDs (%)	Ref.
SPME	GC-MS	6~50	80.4~105	N.A.	[35]
LLE	GC-ECD	1~6	90~160	4.3~12.8	[36]
LLE	GC-ECD	39~67	N.A.	2.1~19.3	[37]
LLE	GC-ECD	8~35	81~106	0.3~2.5	[38]
LLE	GC-MS	15~100	83~126	3~16	[39]
DLLME	PTV-GC-MS	0.4~13.2	79.3~105	<10.2	[40]
DLLME	GC-MS	220~340	96.8~120	4.0~21.3	[41]
HF-LPME	GC-μECD; GC-MS	17~79	92~103	3~16	[42]
SPE	HPLC	600~1 600	97~116	0.2~4.6	[43]
SPME	GC-MS	2~160	N.A.	4~5	[44]
SPME	GC-MS	0.1~3.4	79~110	2~9	[45]
HS	GC-μECD	50	92~102	3.2~3.9	[46]
HS	GC-MS	1020~2520	75.9~94.1	3.6~6.2	[47]
HS	GC-ECD	5~200	66~111	2.1~8.6	[48]
SPE	GC-XSD	N.A.	23~71	0.4~10.3	[49]

Note: N.A. = not available.

## Data Availability

The original contributions presented in this study are included in the article. Further inquiries can be directed to the corresponding authors.
